# Non-Invasive Risk Prediction Based on Right Ventricular Function in Patients with Pulmonary Arterial Hypertension

**DOI:** 10.3390/jcm10215130

**Published:** 2021-10-31

**Authors:** Vazhma Qaderi, Jessica Weimann, Lars Harbaum, Benedikt N. Schrage, Dorit Knappe, Jan K. Hennigs, Christoph Sinning, Renate B. Schnabel, Stefan Blankenberg, Paulus Kirchhof, Hans Klose, Christina Magnussen

**Affiliations:** 1 Department of Cardiology, University Heart & Vascular Centre Hamburg, 20246 Hamburg, Germany; j.weimann@uke.de (J.W.); b.schrage@uke.de (B.N.S.); d.knappe@uke.de (D.K.); c.sinning@uke.de (C.S.); r.schnabel@uke.de (R.B.S.); s.blankenberg@uke.de (S.B.); p.kirchhof@uke.de (P.K.); c.magnussen@uke.de (C.M.); 2 Department of Epidemiology and Preventive Medicine, School of Public Health and Preventive Medicine, Monash University, Melbourne, VIC 3004, Australia; 3 Department of Pulmonology, University Medical Centre Hamburg-Eppendorf, 20246 Hamburg, Germany; l.harbaum@uke.de (L.H.); j.hennigs@uke.de (J.K.H.); klose@uke.de (H.K.); 4 Centre for Pulmonary Arterial Hypertension Hamburg, Martin Zeitz Centre for Rare Diseases, University Medical Centre Hamburg-Eppendorf, 20246 Hamburg, Germany; 5 German Centre for Cardiovascular Research (DZHK), Partner Site Hamburg/Kiel/Luebeck, 20246 Hamburg, Germany; 6 Institute of Cardiovascular Sciences, University of Birmingham, Birmingham B15 2TT, UK

**Keywords:** pulmonary arterial hypertension, risk score, echocardiography, tricuspid annular plane systolic excursion, fractional area change

## Abstract

Background: Right ventricular dysfunction is a major determinant of outcome in pulmonary arterial hypertension (PAH). We aimed to identify echocardiographic right heart parameters associated with adverse outcome and to develop a non-invasive, echocardiography-based risk score for PAH patients. Methods and Results: In 254 PAH patients we analyzed functional status, laboratory results, and echocardiographic parameters. We included these parameters to estimate all-cause death or lung transplantation using Cox regression models. The analyses included a conventional model using guideline-recommended variables and an extended echocardiographic model. Based on the final model a 12-point risk score was derived, indicating the association with the primary outcome within five years. During a median follow-up time of 4.2 years 74 patients died or underwent lung transplantation. The conventional model resulted in a C-Index of 0.539, whereas the extended echocardiographic model improved the discrimination (C-index 0.639, *p*-value 0.017). Ultimately, the newly developed risk score included WHO functional class, 6-min walking distance, N-terminal brain natriuretic peptide concentrations, pericardial effusion, right atrial area, tricuspid annular plane systolic excursion, and fractional area change. Conclusion: Integrating right heart function assessed by echocardiography improves prediction of death or lung transplantation in PAH patients. Independent validation of this finding is warranted.

## 1. Introduction

Pulmonary arterial hypertension (PAH) is a rare and severe chronic lung disease, leading to right heart failure and premature death [1,2]. Although novel therapeutic interventions improved clinical management in the past 20 years, PAH is still associated with high morbidity and mortality [3,4,5]. Precise and individual risk assessment is therefore essential to identify patients at risk and to modify treatment according to disease severity and prognosis.

Comprehensive risk assessment tools have been published in the last years, including a variety of clinical, functional, and hemodynamic variables [1,6,7,8,9]. The 2015 ESC/ERS guidelines for pulmonary hypertension (PH) introduced a risk evaluation tool to predict mortality at one year, allowing a classification of individuals in low (<5%), intermediate (5–10%), and high risk (>10%) categories [1]. Risk estimation in this model is based on clinical deterioration, functional lung capacity, brain natriuretic peptide (BNP), or N-terminal-proBNP (NT-proBNP) concentrations, invasive hemodynamic variables, and echocardiographic evaluation of right atrial (RA) area and pericardial effusion. Due to the required broad clinical and invasive assessment for the ESC/ERS risk score, further registry studies like COMPERA, SPAHR, or the French-registry tried to develop a simplified version of this risk tool, reducing the number of predictors from 12 to eight or less [7,8,9]. In contrast, the REVEAL-Study proposed a risk equation with additional variables, including vital signs and renal impairment to predict adverse outcome in PAH patients [6].

Right ventricular (RV) dysfunction is a major determinant of outcome in PAH [10,11], but echocardiographic parameters of RV function are poorly represented by these risk models [12]. Reduced tricuspid annular plane systolic excursion (TAPSE) was adversely associated with survival in PAH [13,14,15]. Further parameters such as RA dilatation and pericardial effusion were also shown to predict prognosis in PAH [16,17]. Likewise, right longitudinal peak strain was identified as a predictor for all-cause mortality in PAH patients [18,19]. Relatively small sample sizes, limited follow-up, and analysis of mixed populations, including PAH as well as other forms of PH, are limitations of the available data [13,16].

The integration of right ventricular parameters into risk estimation in patients with PAH was identified as an opportunity to improve care by experts [20,21]. We therefore studied the prognostic value of echocardiographic parameters of right heart dysfunction in a uniformly characterized, contemporary clinical cohort of patients with PAH.

## 2. Methods

### 2.1. Study Population

In a retrospective cohort study, we consecutively included 254 patients diagnosed with PAH who presented to the outpatient clinic at the University Medical Center Hamburg-Eppendorf between 2010 and 2018. Diagnosis of PAH was based on current guidelines and resulted from right heart catheterization, requiring mean pulmonary arterial pressure ≥25 mm Hg and pulmonary arterial wedge pressure ≤15 mm Hg [1,22]. The cohort consisted of patients with prevalent disease and newly diagnosed patients. Patients aged under 18 years were excluded from further analyses.

Clinical, laboratory, pulmonary function, and echocardiographic data were collected. Clinical assessment comprised demographics, vital parameters, body mass index (BMI), and PAH-specific medication. Laboratory measurements included NT-proBNP, serum creatinine, and estimated glomerular filtration rate (eGFR) values. Functional assessment contained WHO functional class, a standardized 6-min walk test, and pulmonary function testing, including lung diffusion capacity for carbon monoxide (DLCO) and forced vital capacity (FVC).

According to the local regulations, written informed consent was not necessary for retrospective data collection (§ 12 HmbKHG, ethics review number: 2021-300071-WF), however all data were anonymized and compiled according to the requirements of the local ethics committee and the Declaration of Helsinki. The data underlying this article will be shared on reasonable request to the corresponding author.

### 2.2. Echocardiography

Transthoracic echocardiograms were performed at baseline by experienced examiners using a Philips echocardiography machine (EPIQ 7C, Philips, Eindhoven, The Netherlands). Image analyses were conducted by dedicated software (Tomtec Image Arena version 4.6, Tomtec Imaging Systems, Unterschleissheim, Germany). A second investigator evaluated the results for accuracy to enhance validity of the results. Echocardiographic variables comprised RA area, RV chamber diameters (basal, midventricular, longitudinal), peak tricuspid velocity, vena cava inferior (VCI) diameter, and pericardial effusion. RV function was evaluated by measuring fractional area change (FAC), TAPSE, and 2-dimensional RV speckle-tracking strain. To obtain accurate 2-dimensional speckle-tracking analyses, we only used images with a frame rate≥50 frames per second and sufficient demarcation of the RV free wall [23]. For the measurement of longitudinal free wall strain, the region of interest was manually adjusted and measured in the basal, midventricular, and apical segments of the RV free wall. The myocardial shortening was calculated as the average of the 3 segments [24].

### 2.3. Outcome

The primary endpoint was a composite of all-cause death and lung transplantation in the mid-term follow-up, with censoring after lung transplantation. All patients were regularly assessed in our outpatient department every 3 to 6 months and followed-up for the occurrence of the endpoints via the electronic patients record system of the hospital. Patients were excluded from the analyses if they presented as in need of urgent care with a life expectancy of less than one month.

### 2.4. Statistical Methods

Continuous variables were described as mean ± standard deviation (SD), if normally distributed or median (25th percentile, 75th percentile), if non-normally distributed and categorical variables as absolute numbers (percentages). Sex differences were examined using ANOVA, Mann-Whitney U, or χ2 test. We performed complete case analyses (Table S1).

Univariable analyses were performed to investigate the association of clinical, laboratory, functional, and echocardiographic parameters with the primary outcome. To explore the predictive value of echocardiographic parameters, we performed multivariable Cox regression analyses, adjusting for established risk predictors from the 2015 ESC/ERS guidelines for PH [1]. These comprised WHO functional class, 6-min walk distance (6MWD), NT-proBNP level, as well as pericardial effusion and RA area. Additional echocardiographic parameters from the univariable analyses were added to the multivariable models when assumed statistically significant (*p*-value of <0.05). In sensitivity analyses we also adjusted for all other significant clinical variables from the univariable model.

Based on this, we derived four different multivariable prediction models: A conventional model (Model 1) only including established predictors used in the 2015 ESC/ERS guidelines for PH, an echocardiographic model (Model 2) adding further echocardiographic parameters, and two clinical models (Models 3 and 4) adding other clinical predictors that were significant in the univariable analyses. Hazard ratios (HR) and confidence intervals (CI) for each predictor were given. To compare each model, C-indices corrected by 5-fold cross validation were calculated.

For the purpose of developing a point-based risk score, we assigned continuous variables to categories with pre-defined cut-off values. These cut-off values were adapted either to the intermediate and high-risk categories from the 2015 ESC/ERS guidelines for PH or to prior literature [1,24,25], and were defined as RA area >18 cm, FAC < 35%, TAPSE < 18 mm. WHO functional classes were categorized as WHO I/II, III, and IV. For 6MWD, a cutoff of <400 m was defined. NT-proBNP concentrations were categorized at a threshold of >300 pg/mL and eGFR at a value of <60 mL/min/1.73 m^2^. Systolic blood pressure had a cutoff of <110 mmHg and DLCO was categorized at <40%. In addition, we compared the performance of these expert recommended cut-off values with the data-driven calculated cut-off values (Table S2). Based on the ß-coefficients from the multivariable Model 2, a point-based risk score was developed using the approach of the Framingham Study [26]. This score allowed the estimation of the individual risk for all-cause mortality or lung transplantation within 5 years. The point score was calculated for every patient and fitted as spline with 4 degrees of freedom for the event [27]. The results were plotted with the relative event rate on y-axis referencing patients with zero points.

A *p*-value of <0.05 was considered statistically significant. Statistical analysis was performed using the software R Version 4.0.3 (10 October 2020).

## 3. Results

### 3.1. Patient Characteristics

Demographic features, clinical characteristics, and functional parameters are displayed in Table 1. Of 254 patients included in the analyses, 33.9% were female and the median age was 65.5 years (49.4, 74). Systolic blood pressure was 128 mmHg (SD ± 22.8). Most patients were in WHO functional class III or IV and had a severely reduced 6MWD (269.2 m, SD ±183.9). Pulmonary function testing showed a DLCO of 58% (38.9, 77) and a FVC of 75% (58, 86). The majority of patients had elevated NT-proBNP concentrations >300 pg/mL and 39% showed renal impairment with eGFR of less than 60 mL/min/1.73 m^2^. In 23.2% patients no PAH-specific treatment was established, 48% received a monotherapy, and 28.7% had a combination therapy with 2 or more different drugs. Among these patients, 45% had arterial hypertension, 16.5% were diagnosed with diabetes, 20.5% had preexisting coronary artery disease, and 3.6% had peripheral artery disease.

### 3.2. Echocardiographic Parameters

Echocardiographic parameters are presented in Table 2. The majority of patients had an enlarged RA area and showed notably enlarged RV base and RV medial dimensions compared to reference values [24]. The longitudinal diameter of the RV was within the normal range. Pericardial effusion was detected in 14 individuals. RV function was impaired in the majority of the cohort shown by reduced FAC, reduced TAPSE, and impaired RV free wall strain.

### 3.3. Regression Analysis

**Figure 1:**
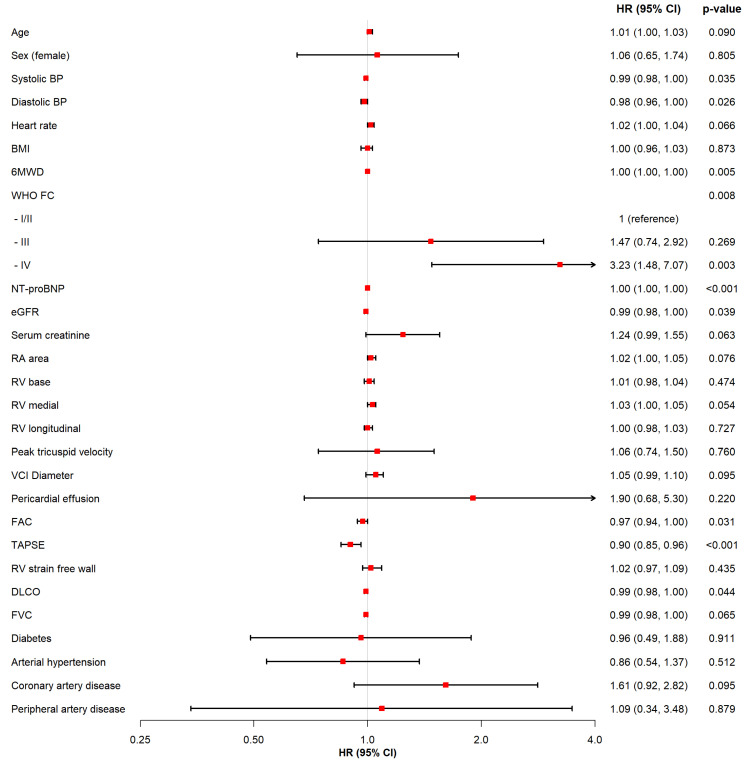
Predictors of death or lung transplantation in univariable Cox regression analyses. Legend: This figure displays the association of each variable with all-cause mortality and lung transplantation based on univariable Cox regression analyses. Abbreviations: HR = Hazard Ratio, CI = Confidence Interval, No. = Number, BP = blood pressure, BMI = body-mass-index, 6MWD = 6-min walk distance, FC = functional class, NT-proBNP = N-terminal-pro hormone brain peptide, eGFR = estimated glomerular filtration rate, RA = right atrial, RV = right ventricular, VCI = vena cava inferior, FAC = fractional area change, TAPSE = tricuspid annular plane systolic excursion, DLCO = diffusing capacity of the lungs for carbon monoxide, FVC = forced vital capacity.

During a median follow up time of 4.18 years, 63 (24.8%) patients died and 11 (4.3%) underwent lung transplantation, resulting in an incidence rate of the composite endpoint of 25.3 per 1000 person-years. In univariable Cox regression analyses, the following parameters were associated with the composite endpoint: low systolic blood pressure, WHO functional class IV, lower 6MWD, NT-proBNP concentrations, renal impairment, impacted DLCO, reduced TAPSE, and reduced FAC (Figure 1).

Based on the above defined criteria TAPSE, FAC, eGFR, systolic blood pressure, and DLCO were selected as additional parameters for inclusion in the multivariable models (Table 3, Table S3).

The conventional model resulted in a C-index of 0.539 (Model 1). When adding TAPSE < 18 mm and FAC < 35% to Model 1, the C-index increased to 0.639 (*p*-value 0.017, Model 2). When further adjusting for eGFR <60 mL/min/1.73 m^2^, systolic blood pressure <110 mmHg, and DLCO < 40%, the C-indices did not improve relevantly, but TAPSE remained an independent predictor for the outcome (Table S3).

### 3.4. Non-Invasive Risk Score

**Figure 2:**
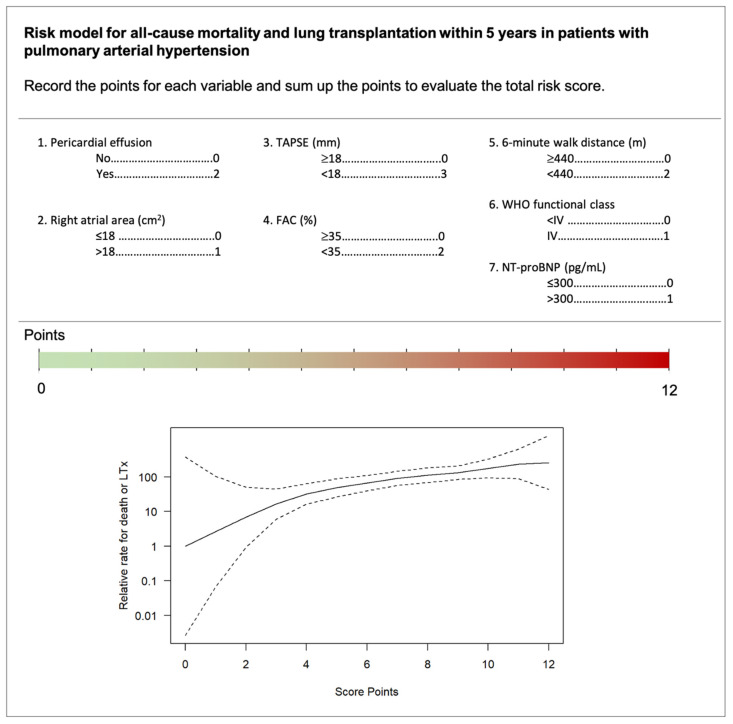
Point-based score to calculate the individual risk of all-cause mortality and lung transplantation within 5 years in patients with PAH. Legend: We developed a point-based score to assess the risk of death or lung transplantation within 5 years in patients with pulmonary arterial hypertension. The score ranges from 0 to 12 points and indicates the relative risk. Abbreviations: TAPSE = tricuspid annular plane systolic excursion, FAC = fractional area change, NT-proBNP = N-terminal-pro hormone brain peptide, LTx = lung transplantation.

Based on the multivariable results from Model 2, each predictor was allocated to a point score (Figure 2). NT-proBNP concentrations >300 pg/mL, WHO functional class IV, and RA area >18 cm^2^ were assigned one point. Presence of pericardial effusion, 6MWD < 440 m and FAC < 35% were given two points. TAPSE <18 mm as main predictor was represented by three points. The points for each variable can be summed up to display the total risk. The final risk score ranges from 0 to 12 points, while higher numbers indicate higher risk.

## 4. Discussion

We demonstrated the clinical value of echocardiographically determined RV parameters in risk prediction of PAH patients. We could (1) emphasize the prognostic value of TAPSE as an independent predictor for all-cause mortality and lung transplantation, (2) underline the importance of FAC, which is not yet included in current guidelines, and (3) develop an easily applicable non-invasive tool for PAH risk assessment.

### 4.1. Cohort and Clinical Risk Parameters

Our study population was predominantly male, with a median age of 65.5 years and a heterogenous PAH etiology. A shift in clinical characteristics of PAH patients was observed in the past decades. While earlier studies reported mainly young female patients, a decrease of the female predominance with age and an increase of the median age at diagnosis has been reported in recent years [28,29]. In this heterogenous but contemporary PAH cohort, we were able to confirm the predictive value of recognized clinical variables such as 6MWD, WHO functional class, and NT-proBNP levels [1], and additionally identify less established variables like renal impairment and DLCO as predictors for adverse outcome in PAH. Results from screening of susceptible subjects, both genetically or due to an underlying condition such as systemic sclerosis, further emphasize the importance of DLCO for early detection of pulmonary vascular disease [30,31]. Overall, our results support findings from the REVEAL-study and highlight the importance of comorbidities and pulmonary function testing in clinical evaluation [6,32].

### 4.2. Echocardiographic Parameters

The 2015 ESC/ERS guidelines for PH limit the use of echocardiographic assessment for risk prediction to RA area and pericardial effusion. In several studies, pericardial effusion was reported to be a predictor of adverse outcome [6,16]. In contrast, we could not confirm its predictive value, which might be explained by the low prevalence of pericardial effusion in our cohort (5.8%) compared to other studies (ranging from 14–53.2%) [16,19,33]. In our study, RA area also could not predict adverse outcome. However, the recommendation to use RA area is based on expert consensus and small or older studies, while RA enlargement mainly reflects late stages of right ventricular dysfunction [16,17]. Data from clinical trials have demonstrated treatment–associated changes in TAPSE and FAC, but not RA. Thus, risk assessment should rather involve parameters reflecting early decline of right heart function than measures of advanced disease [34].

Previous studies showed that RV variables predict risk in patients with PH. TAPSE, being an important RV parameter, showed high reproducibility with small interobserver differences [35]. The 2015 ESC/ERS guidelines for PH consider the use of TAPSE for assessing disease severity (1), but there is evidence that TAPSE also relates to mortality in pre-capillary PH [14,15,19,36]. However, these results are limited by low numbers of events, short follow-up, or missing adjustment for confounders. Furthermore, study populations were often not restricted to patients with PAH, but also included patients with other groups of PH [14,19]. As the pathophysiology, clinical presentation, and treatment options differ substantially within the different PH groups, these findings cannot be generalized to other PH entities [37,38]. In our well characterized PAH cohort, TAPSE predicted adverse prognosis even after adjusting for established confounders and additional predictors, including eGFR, systolic blood pressure, and DLCO; this confirms the robustness of our findings.

Data on FAC as a predictor in PAH is sparse, while FAC is mainly investigated in cardiac magnetic resonance imaging [39]. Fine and colleagues showed that FAC predicts cardiopulmonary death, lung transplantation, or PAH-related hospitalization, while adjustment for confounders was missing [19]. Our data indicate that FAC might have a certain prognostic value, although the association was not significant after multivariable adjustment.

The value of RV strain as measure of RV dysfunction and predictor of adverse outcome in PAH has been identified in previous studies [18,19,40]. There is inconsistent evidence concerning the best cutoff values for RV strain and its relation to disease severity [18,40]. Similar to Sachdev and colleagues, we found a median RV free wall strain of −14.0% in patients with comparable disease stages. However, RV strain did not reliably predict adverse outcome, which could be explained by the reduced number of available RV strain measurements.

### 4.3. Point-Based Risk Score

Application of current risk equations is limited by inclusion of invasive measures, which may not be available in all patients [6,7,8,9]. In contrast, echocardiographic evaluation is easily accessible, collected in a standardized way, and time and cost effective [41]. Adding RV parameters to a conventional model of clinical parameters, RA area and pericardial effusion, significantly improved the diagnostic performance. The C-index of 0.64 is comparable to prior risk models, but in contrast did not require the incorporation of invasive parameters [7,8]. In our model, echocardiographic parameters were assigned the highest point values emphasizing their value in risk prediction, while WHO functional class, NT-proBNP, and RA area received the lowest point values.

In terms of the constant improvement of PAH outcome, prediction of mid-term risk is becoming increasingly important [3]. While previous models usually aimed to predict the one year outcome [1,6,8,9], our results relevantly contribute to the horizon of risk assessment.

### 4.4. Strengths and Limitations

A major strength is the well characterized cohort and the recruitment from a specialized PAH-center. Furthermore, the extensive follow-up and the precise echocardiographic assessment, including novel variables precisely mapping RV architecture and function, mark up our cohort. However, the results are limited by the retrospective nature and by representing a single-center design. Therefore, external validation of our model is necessary to confirm our findings. Furthermore, change in therapeutic strategies during the long recruitment period from 2010 to 2018 and including prevalent and newly diagnosed patients might have influenced outcome measures, but concurrently allowed us to report a rather long follow-up and reflects clinical reality. Due to the dependence on image quality for speckle tracking echocardiography the number of missing values for RV, free wall strain might have led to an underestimation of the predictive strength of this variable. Therefore, further prospective studies with detailed echocardiographic assessment are necessary to clarify the impact of specific RV echocardiographic parameters for risk prediction.

### 4.5. Conclusions

Our findings highlight the importance of echocardiographic assessment in patients with PAH. An easily applicable point-based score that integrates non-invasive echocardiographic parameters of right heart morphology and function improves the prediction of death or lung transplantation in PAH patients.

## Figures and Tables

**Table 1:** Baseline characteristics of the overall study population.

	All (N = 254)	Females (N = 86)	Males (N = 168)	*p*-Value
**Clinical parameters**				
Age (years)	65.5 (49.9, 74.0)	66.0 (55.9, 75.0)	65.0 (48.0, 74.0)	0.24
Sex (females) No. (%)	86 (33.9)			
PAH etiology No. (%)				
Idopathic	160 (63.7)	57 (66.3)	103 (62.4)	0.64
Heritable	5 (2.0)	1 (1.2)	4 (2.4)	0.84
Drug/toxin induced	2 (0.8)	1 (1.2)	1 (0.6)	1.00
Associated	84 (33.5)	27 (31.4)	57 (34.5)	0.72
Systolic BP (mmHg)	128.0 ± 22.8	127.2 ± 22.9	128.4 ± 22.7	0.70
Diastolic BP (mmHg)	75.5 ± 14.3	75.7 ± 15.9	75.3 ± 13.5	0.85
Heart rate (bpm)	77.3 ± 13.8	75.9 ± 13.0	78.1 ± 14.1	0.22
BMI (kg/m^2^)	27.3 ± 6.1	27.2 ± 5.0	27.4 ± 6.6	0.80
WHO FC: I/II No. (%)	54 (21.3)	22 (25.6)	32 (19.2)	0.31
WHO FC: III No. (%)	162 (64.0)	51 (59.3)	111 (66.5)	0.32
WHO FC: IV No. (%)	37 (14.6)	13 (15.1)	24 (14.4)	1.00
**Functional and laboratory parameters**				
6MWD (m)	269.2 ± 183.9	255.5 ± 203.4	276.2 ± 173.4	0.40
6MWD < 440 m No. (%)	209 (84.3)	67 (80.7)	142 (86.1)	0.37
FVC (%)	75.0 (58.0, 86.0)	77.0 (59.4, 83.0)	73.5 (58.0, 88.0)	0.78
DLCO (%)	58.0 (38.9, 77.0)	53.5 (31.0, 74.2)	60.0 (42.4, 77.0)	0.12
DLCO < 40% No.(%)	50 (25.8)	23 (32.9)	27 (21.8)	0.13
**Laboratory parameter**				
NT-proBNP (pg/mL)	1570.0 (437.3, 4099.8)	1732.0 (461.3, 4464.7)	1509.0 (434.2, 3462.2)	0.56
NT-proBNP > 300 pg/mL No. (%)	201 (80.1)	67 (78.8)	134 (80.7)	0.85
Serum creatinine (mg/dL)	1.0 (0.8, 1.2)	1.2 (1.0, 1.5)	1.0 (0.8, 1.2)	<0.001
eGFR (mL/min/1.73 m^2^)	67.7 (47.3, 93.3)	50.3 (36.5, 63.1)	83.3 (61.4, 101.9)	<0.001
eGFR < 60 mL/min/1.73 m^2^ No. (%)	97 (39.0)	60 (69.8)	37 (22.7)	<0.001
**Medication**				
PAH drugs ≥ 2 No. (%)	73 (28.7)	23 (26.7)	50 (29.8)	0.72
Phosphodiestarase 5 inhibitor No. (%)	136 (53.5)	45 (52.3)	91 (54.2)	0.88
Endothelin receptor antagonist No. (%)	113 (44.5)	38 (44.2)	75 (44.6)	1.00
Prostacyclin agonist No. (%)	31 (12.2)	8 (9.3)	23 (13.7)	0.42
**Pre-existing diseases**				
Arterial hypertension No. (%)	112 (45.0)	41 (47.7)	71 (43.6)	0.63
Diabetes No. (%)	41 (16.5)	19 (22.1)	22 (13.5)	0.12
Coronary artery disease No. (%)	51 (20.5)	26 (30.2)	25 (15.3)	0.009
Peripheral artery disease No. (%)	9 (3.6)	4 (4.7)	5 (3.1)	0.78

Abbreviations: No. = Number, BP = blood pressure, bpm = beats per minute, FC = functional class, 6MWD = 6-min walk distance, FVC = forced vital capacity, DLCO = diffusing capacity of the lungs for carbon monoxide, NT-proBNP = N-terminal pro hormone brain peptide, eGFR = estimated glomerular filtration rate, PAH = pulmonary arterial hypertension, units in brackets, headings in bold.

**Table 2:** Echocardiographic parameters.

Echocardiographic Parameters	All (N = 254)	Females (N = 86)	Males (N = 168)	*p*-Value
RA area (cm^2^)	24.0 (19.0, 29.0)	26.0 (21.0, 31.3)	23.0 (19.0, 29.0)	0.049
RA area > 18 cm^2^ No. (%)	190 (79.5)	67 (82.7)	123 (77.8)	0.48
RV base (mm)	46.5 (41.0, 52.0)	49.0 (43.7, 54.0)	45.0 (40.0, 51.0)	0.003
RV medial (mm)	37.0 (30.0, 43.0)	38.0 (30.4, 43.0)	36.5 (30.0, 42.1)	0.32
RV longitudinal (mm)	70.0 (64.0, 77.0)	74.0 (68.0, 82.0)	68.0 (61.0, 74.1)	<0.001
Peak tricuspid velocity (m/s)	4.0 (3.0, 4.0)	4.0 (3.0, 4.0)	4.0 (3.0, 4.0)	0.28
VCI diameter (mm)	18.0 (15.0, 21.0)	20.0 (17.0, 22.8)	17.0 (14.0, 21.0)	0.001
Pericardial effusion No. (%)	14 (5.8)	5 (6.1)	9 (5.7)	1.00
FAC (%)	26.0 (20.0, 33.1)	26.0 (19.4, 31.6)	27.0 (20.0, 35.0)	0.43
FAC < 35 No. (%)	159 (77.2)	56 (82.4)	103 (74.6)	0.29
TAPSE (mm)	17.0 (14.0, 21.0)	17.0 (14.0, 21.0)	17.0 (14.0, 21.0)	0.69
TAPSE < 18 mm No. (%)	124 (53.7)	46 (57.5)	78 (51.7)	0.48
RV strain free wall (%)	−14.0 (−18.0, −10.0)	−13.5 (−18.0, −10.0)	−14.0 (−17.3, −10.7)	1.00

Abbreviations: RA = right atrial, RV = right ventricular, VCI = vena cava inferior, FAC = fractional area change, TAPSE = tricuspid annular plane systolic excursion, units in brackets, headings in bold.

**Table 3:** Multivariable Cox regression models predicting all-cause mortality and lung transplantation.

	Model 1			Model 2		
	HR (95% CI)	HR per SD (95% CI)	*p*-Value	HR (95% CI)	HR per SD (95% CI)	*p*-Value
WHO FC I/II	1 (reference)			1 (reference)		
WHO FC III	1.01 (0.46, 2.23)	1.00 (0.69, 1.46)	0.983	0.91 (0.36, 2.28)	0.96 (0.62, 1.47)	0.836
WHO FC IV	1.74 (0.68, 4.43)	1.21 (0.88, 1.68)	0.244	1.54 (0.52, 4.58)	1.16 (0.80, 1.69)	0.437
NT-proBNP > 300 pg/mL	1.69 (0.77, 3.71)	1.24 (0.90, 1.70)	0.188	1.38 (0.59, 3.21)	1.14 (0.80, 1.63)	0.461
6MWD < 440 m	2.08 (0.86, 5.02)	1.31 (0.95, 1.82)	0.103	1.77 (0.73, 4.31)	1.24 (0.89, 1.73)	0.210
RA area > 18 cm^2^	1.68 (0.81, 3.48)	1.23 (0.92, 1.66)	0.160	1.33 (0.57, 3.10)	1.13 (0.79, 1.60)	0.508
Pericardial effusion	1.53 (0.54, 4.36)	1.11 (0.86, 1.43)	0.425	1.82 (0.63, 5.31)	1.15 (0.90, 1.48)	0.271
TAPSE < 18 mm				2.36 (1.28, 4.35)	1.54 (1.13, 2.09)	0.006
FAC < 35%				1.56 (0.67, 3.64)	1.21 (0.84, 1.73)	0.301
N	225			189		
N events	62			52		
C-Index	0.539 (0.446, 0.632)			0.639 * (0.550, 0.729)		

Model 1: includes established parameters: WHO functional class, NT-proBNP, 6 MWD, RA area, and pericardial effusion. Model 2: includes predictors from Model 1 and the echocardiographic parameters TAPSE and FAC * *p*-value when compared to Model 1 = 0.017, Abbreviations: HR = Hazard Ratio, CI = Confidence Interval, SD = Standard deviation, FC = functional class, NT-proBNP = N-terminal-pro hormone brain peptide, 6MWD = 6-min walk distance, RA = right atrial, TAPSE = tricuspid annular plane systolic excursion, FAC = fractional area change.

## Data Availability

The data underlying this article will be shared on reasonable request to the corresponding author.

## Supplementary Materials

The following are available online at https://www.mdpi.com/article/10.3390/jcm10215130/s1. Table S1: Missing values of the overall study population. Table S2: Comparison between expert recommended and generated cut-offs for survival analysis (univariate). ROC analysis at year 5. C-Index is bootstrap corrected (B = 1000). Table S3: Multivariable Cox regression models predicting all-cause mortality and lung transplantation shown as sensitivity analyses adjusting for all significant clinical variables.
