# Supplementary material for: Non-Invasive Risk Prediction Based on Right Ventricular Function in Patients with Pulmonary Arterial Hypertension
Source: J Clin Med. 2021 Oct 31;10(21):5130. doi: 10.3390/jcm10215130 (PMC8584811; doi:10.3390/jcm10215130)
Supplement: Supplementary file 1 [file jcm-10-05130-s001.zip › jcm-1408906-supplementary.pdf]

Supplementary Material

Table S1: Missing values of the overall study population

	Missing value (N)
Age	0/254
Sex	0/254
PAH etiology	3/254
Systolic BP	5/254
Diastolic BP	5/254
Heart rate	4/254
BMI	1/254
WHO FC	1/254
6MWD	6/254
FVC	22/254
DLCO	60/254
NT-proBNP	3/254
Serum creatinine	5/254
eGFR	5/254
Phosphodiestarase 5 inhibitor	0/254
Endothelin receptor antagonist	0/254
Prostacyclin agonist	0/254
Arterial hypertension	5/254
Diabetes	5/254
Coronary artery disease	5/254
Peripheral artery disease	5/254
RA area	15/254
RV base	18/254
RV medial	20/254
RV longitudinal	20/254
Peak tricuspid velocity	54/254
VCI diameter	65/254
Pericardial effusion	13/254
FAC	48/254
TAPSE	23/254
RV strain free wall	113/254

Abbreviations: PAH = pulmonary arterial hypertension, BP = blood pressure, BMI = body-mass-index, FC = functional class, 6MWD = 6-minute walk distance, FVC = forced vital capacity, DLCO = diffusing capacity of the lungs for carbon monoxide, NT-proBNP = N-terminal-pro hormone brain peptide, eGFR = estimated glomerular filtration rate, RA = right atrial, RV = right ventricular, VCI = vena cava inferior, FAC = fractional area change, TAPSE = tricuspid annular plane systolic excursion.

Table S2: Comparison between expert recommended and generated cut-offs for survival analysis (univariate). ROC analysis at year 5. C-Index is bootstrap corrected (B=1000).

	Cut-off	Sens (%)	Spec (%)	PPV (%)	NPV (%)	C-Index
NT-proBNP Calculated	2288	60.95	75	58.29	77.02	0.62
NT-proBNP Expert	300	91.55	32.81	43.85	87.14	0.58
6MWD Calculated	320	38.56	39.06	26.99	52.12	0.6
6MWD Expert	440	8.72	75	16.93	58.45	0.57
RA area Calculated	26	47.34	72.13	45.24	73.79	0.58
RA area Expert	18	86.18	24.59	35.73	78.54	0.55
TAPSE Calculated	17	29.09	45.9	20.89	56.85	0.63
TAPSE Expert	18	21.62	52.46	18.26	57.67	0.62
FAC Calculated	28	38.22	62.5	30.84	69.8	0.57
FAC Expert	35	15.31	78.57	23.82	67.95	0.54

Abbreviations: Sens = Sensitivity, Spec = Specificity, PPV = positive predictive value, NPV = negative predictive value, NT-proBNP = N-terminal-pro hormone brain peptide, 6MWD = 6-minute walk distance, RA = right atrial, TAPSE = tricuspid annular plane systolic excursion, FAC = fractional area change.

Table S3: Multivariable Cox regression models predicting all-cause mortality and lung transplantation shown as sensitivity analyses adjusting for all significant clinical variables

	Model 3			Model 4		
	**HR (95% CI)**	**HR per SD (95% CI)**	**p-value**	**HR (95% CI)**	**HR per SD (95% CI)**	**p-value**
WHO FC I/II	1 (reference)			1 (reference)		
WHO FC III	0.89 (0.35, 2.24)	0.95 (0.61, 1.46)	0.805	0.69 (0.21, 2.25)	0.85 (0.50, 1.44)	0.538
WHO FC IV	1.39 (0.46, 4.17)	1.12 (0.76, 1.65)	0.556	0.70 (0.15, 3.26)	0.90 (0.56, 1.44)	0.654
NT-proBNP >300pg/mL	1.34 (0.57, 3.17)	1.13 (0.79, 1.60)	0.503	1.34 (0.50, 3.64)	1.13 (0.75, 1.69)	0.561
6MWD <440m	1.78 (0.73, 4.35)	1.24 (0.89, 1.72)	0.206	1.22 (0.47, 3.19)	1.08 (0.75, 1.54)	0.682
RA area >18cm^2	1.32 (0.56, 3.15)	1.12 (0.79, 1.60)	0.526	2.93 (0.87, 9.90)	1.55 (0.94, 2.54)	0.083
Pericardial effusion	1.58 (0.54, 4.60)	1.12 (0.86, 1.44)	0.399	2.24 (0.70, 7.21)	1.22 (0.91, 1.64)	0.175
TAPSE <18mm	2.32 (1.27, 4.27)	1.53 (1.13, 2.07)	**0.006**	2.70 (1.29, 5.65)	1.64 (1.13, 2.38)	**0.009**
FAC <35%	1.68 (0.71, 4.00)	1.25 (0.86, 1.80)	0.240	1.09 (0.40, 2.95)	1.04 (0.68, 1.58)	0.870
eGFR <60mL/min/1.73m^2	1.70 (0.95, 3.05)	1.30 (0.98, 1.72)	0.073	2.12 (1.05, 4.29)	1.45 (1.02, 2.05)	**0.036**
Systolic BP <110mmHg	1.52 (0.78, 2.96)	1.18 (0.91, 1.54)	0.219	1.96 (0.89, 4.32)	1.30 (0.95, 1.76)	0.096
DLCO <40%				2.83 (1.25, 6.40)	1.56 (1.10, 2.20)	**0.013**
N	182			149		
N events	52			39		
C-Index	0.659[#]			0.667[*]		

Model 3: includes established parameters (WHO functional class, NT-proBNP, 6MWD, RA area, pericardial effusion), additional echocardiographic variables (TAPSE, FAC) and clinical and laboratory variables (systolic BP and eGFR)
Model 4: includes predictors from Model 3 and the pulmonary function testing variable DLCO
[#]p-value when compared to Model 2 = 0.58**,** *p-value when compared to Model 2 = 0.52, Abbreviations: HR = Hazard Ratio, CI = confidence interval, SD = Standard deviation, FC = functional class, NT-proBNP = N-terminal-pro hormone brain peptide, 6MWD = 6-minute walk distance, RA = right atrial, TAPSE = tricuspid annular plane systolic excursion, FAC = fractional area change, eGFR = estimated glomerular filtration rate, BP = blood pressure, DLCO = diffusing capacity of the lungs for carbon monoxide.
